# Determination of bioavailable phosphorus in water samples using bioassay methods

**DOI:** 10.1016/j.mex.2020.100807

**Published:** 2020-02-20

**Authors:** Marcel L. Dijkstra, Martin T. Auer, Anika Kuczynski, Renn Lambert

**Affiliations:** a Department of Engineering Technology, University of Wisconsin Oshkosh, 800 Algoma Boulevard Oshkosh, WI 54901, USA; b Department of Civil and Environmental Engineering, Michigan Technological University, 1400 Townsend Drive, Houghton, MI 49931, USA; c National Institute of Water and Atmospheric Research, 10 Kyle St., Riccarton, Christchurch 8011, New Zealand; d LimnoTech Inc., 501 Avis Dr., Ann Arbor, MI 48108, USA

**Keywords:** Bioavailable phosphorus, Bioassay, Limiting nutrient

## Abstract

The total phosphorus analyte (TP) has a long history of use in monitoring and regulatory applications relating to management of cultural eutrophication in freshwaters. It has become apparent, however, that the fraction of the TP analyte ultimately available to support algal growth varies significantly spatially (within a system), seasonally, and among systems. The algal bioassay methods described here provide an approach for determining the bioavailable fraction of the three operationally defined components of TP: soluble reactive phosphorus (SRP), dissolved organic phosphorus (DOP), and particulate phosphorus (PP) in effluents and tributaries discharging to lakes and reservoirs. Application of the technique facilitates a quantitative ranking and targeting of bioavailable phosphorus sources for management.

• One congruent method to fractionate particulate and soluble phosphorus (found in aquatic samples) into bioavailable and unavailable fractions was developed based on compilation, adaptation and expansion of two methods from the late 1970s and early 1980s.
• Detailed descriptions for culturing phosphorus-starved algae, sub-sampling schedules, kinetics determination, and data presentation are provided
• Reproducibility is demonstrated by replication and closure of a mass balance on phosphorus.

## Graphical abstract

**Figure:**
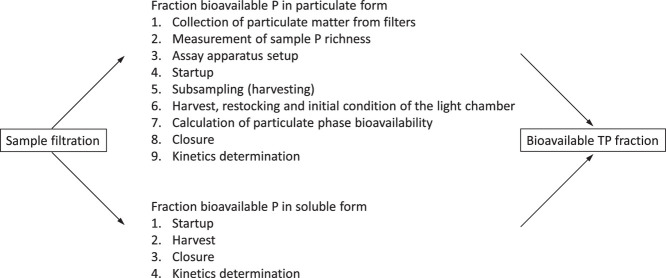

**Table:** Specifications Table

Subject area:	Environmental Science
More specific subject area:	Fractionation of phosphorus in aquatic matrices
Method name:	Bioavailability and kinetics determination of phosphorus constituents in aquatic matrices
Name and reference of original method:	(1) Soluble phase: bottle test procedure [13]. (2) Particulate phase: dual culture diffusion apparatus (DCDA) method [7]*.*
Resource availability:	*If applicable, include links to resources necessary to reproduce the method (*e.g., *data, software, hardware, reagent)*

## Introduction

Phosphorus (P) is often the growth-limiting nutrient for algae in freshwaters [14] and thus the appropriate target for management of eutrophication and its attendant manifestations. Eutrophication is typically managed by treatment of its symptoms (e.g., aeration for oxygen depletion), control of phosphorus inputs, or a combination of the two approaches [6]. The U.S. Clean Water Act (Section 303d) provides for determination of a total maximum daily load (TMDL) as a starting point where management of compromised systems is sought through control of phosphorus inputs. The required loading reduction, i.e., to meet the TMDL, is often set using mathematical models [5]. Historically, the managed phosphorus metric has been total phosphorus (TP) concentration. It has become apparent, however, that the TP analyte includes several moieties, some of which are available to support algal growth (bioavailable) and some of which are not (refractory) [4]. Thus, two loading sources delivering identical TP loads could have widely differing impacts on water quality because of differences in their bioavailability [see 9,10]. In some cases, where control options target a more refractory P fraction, manifestations of eutrophication can increase even as TP loads decrease [3].

The concept of P bioavailability is grounded in the operational definition of total phosphorus as having three components: soluble reactive phosphorus (SRP), dissolved organic phosphorus (DOP), and particulate phosphorus (PP). Soluble reactive phosphorus is ~100% bioavailable, while DOP and PP have smaller bioavailable fractions. The bioavailable P content of a sample varies among sources (e.g., wastewater effluents and tributary streams) and within (spatially and seasonally) and among systems. Phosphorus bioavailability may be estimated using chemical fractionation or algal bioassay techniques. Each approach offers advantages and disadvantages. For example, the chemical fractionation technique is readily adaptable to an automated analytical program but does not determine the bioavailable fraction of DOP or the extractable biogenic fraction of PP; thus, chemical fractional potentially leads to underestimates of the overall P bioavailability of the sample. The algal bioassay approach, although potentially more labor-intensive, provides a direct determination of the ability of phosphorus forms to support algal growth [12]. Further, the algal bioassay technique permits characterization of the rate at which bioavailable P is made manifest from DOP and PP.

Here, the details of the bioassay procedures for determination of the fraction bioavailable of SRP, DOP and PP in effluents and tributary samples are provided, yielding an aggregate bioavailable fraction that may be expressed in terms of TP for use in prioritizing management strategies. The method described here is a compilation, adaptation and expansion of two methods from the late 1970s [13] and early 1980s [7] which were synthesized into one congruent method to fractionate particulate and soluble phosphorus. Replicate assay measurements resulted in a coefficient of variation of 7.7% or better and a mass balance closure (P_bioavailable_ = P_added_ − P_remaining_) of over 90% was achieved.

### Physical – chemical analyses

• Dry Mass (DM, mg): determined by placement of sample (particulate on 0.45 µm membrane after filtration) in drying oven at 103–105 °C until dry, [1; 2540 B]
• Soluble reactive phosphorus (SRP): determined by the ascorbic acid method [1; 4500-P E] on sample filtrate after filtration with a 0.45 µm membrane filter
• Total dissolved phosphorus (TDP): determined by ammonium persulfate digestion [1; 4500-P B] on sample filtrate after filtration with a 0.45 µm membrane filter followed by the ascorbic acid method [1; 4500-P E] on the digested sample
• Dissolved organic phosphorus (DOP): calculated by difference (DOP = TDP–SRP)
• Particulate phosphorus (PP): determined by ammonium persulfate digestion [1; 4500-P B] on the filtride (suspended solids remaining on a 0.45 µm membrane filter after filtration) followed by the ascorbic acid method [1; 4500-P E] on the digested sample
• Total phosphorus (TP): determined by ammonium persulfate digestion [1; 4500-P B] on sample without filtration followed by the ascorbic acid method [1; 4500-P E] on the digested sample

### Algal culture

Algal culture species: *Selenastrum capricornutum*

Algal culture development and maintenance

(1) Macronutrient stock solutions:
  A: Combine 25.5 g of NaNO_3_, 15.0 g of NaHCO_3_, 14.7 g of MgSO_4_⋅7H_2_O, 12.2 mg MgCl_2_⋅6H_2_O and 4.41 g of CaCl_2_⋅2H_2_O in 1 L of distilled, deionized water.
  B: Keep a separate stock solution for 0.813 g of K_2_HPO_4_ in 1 L of distilled, deionized water.
  C: Create a stock solution for P-free algal growth medium with 0.894 g KCl in 1 L of distilled, deionized water (to be added instead of the K_2_HPO_4_ for P-free algal growth medium).
(2) Trace metal-EDTA stock solution: combine 0.186 g of H_3_BO_3_, 0.414 g MnCl2⋅4H_2_O, 3.27 g ZnCl_2_, 1.43 g CoCl_2_⋅6H_2_O, 7.26 g Na_2_MoO_4_⋅2H_2_O, 9.60 g FeCl_3_ and 0.300 g NaEDTA⋅2H_2_0 in 1 L of distilled, deionized water.
(3) Store growth media in amber bottles at 4 °C.
(4) Add 1 mL of macronutrient stock solution A and B (with P) and 1 mL of trace metal-EDTA stock solution to 750 mL of distilled, deionized water and dilute to 1 L to create algal growth medium in a glass flask (e.g., large Erlenmeyer, beaker, or bulb growth flask). Cover the glassware containing the growth medium with aluminum foil and autoclave. Also autoclave glass and/or rubber tubing to be used to aerate the culture in the flask. Let the flask and tubing cool to room temperature prior to adding a *Selenastrum capricornutum* inoculant (~ 250 mL from a culture that has grown for 7–10 days in a total 3L of algae growth medium). The volume of the culture should not exceed half the volume of the container. The mixture is aerated using a bubbler and glass wool as a flask plug and filter to prevent bacteria and other particulates from contaminating the culture.
(5) Maintain the culture for 3 weeks at saturating light conditions and aeration but without additional nutrients. The culture will “green up” and become P-limited (starved) after 3 weeks and is usable as an inoculum for 2 weeks, after which a new P-starved culture needs to be used. Older algae cultures may not have enough viable cells to conduct experiments.

### Pre-sampling

The soluble phase bioassay requires a minimum 2 L water sample to ensure that there is enough volume for replicate assay sub-sampling over the course of the incubation. Similarly, the particulate phase bioassay requires at least 500 and optimally 1000 µg PP. To determine how much of the original water sample must be filtered to achieve the target PP mass for the particulate assay, the water is pre-sampled for its PP concentration ($\frac{\mu gP}{L}$). This permits calculation of the water volume to be filtered:

(1) $$\mathrm{Volu}\mathrm{me}\mathrm{to}\mathrm{be}\mathrm{Filt}\mathrm{ered}\left(L\right)=\frac{1000\left(\mu \mathrm{gP}\right)}{PP_{\mathrm{samp}\mathrm{le}}\;\left(\mu gP/L\right)}$$

Note that pre-sampling for PP concentration is intended to provide guidance only for the volume of sample to be collected and subsequently filtered to yield the amount of PP required for the assay. Additional PP analyses are run during *Startup, Harvesting* and *Closure* to track bioavailability and perform a mass balance.

### Sample filtration

The water sample to be assayed is filtered to obtain subsamples for the soluble phase (filtrate) and particulate phase (filtride) assays. Several liters (the minimum amount to be filtered is estimated with Eq. (1)) of water will typically need to be collected for particulate phase assays. Thus, the use of a high-pressure filtration apparatus (142 mm diameter with a membrane filter, e.g., Millipore Express PLUS with 0.45 µm filter at a pressure not to exceed 30 kPa) is recommended. The filters need to be sufficiently loaded with particulate so that it can be collected by scraping the filter. Multiple filters may be utilized, changing filters as the flow rate is reduced to a slow drip. It is worthwhile to introduce the sample in batches with smaller volumes added as time progresses and particulate matter accumulates on a filter; this prevents large unfiltered water volumes from collecting in the cylinder on the filter when the flow rate declines to a drip. Retain an absolute minimum of 2 L of filtrate for a single soluble bioassay (respectively more volume for replicate soluble assays) and, if not used immediately, store in the dark at 4 °C with a maximum holding time of 24 hrs. Store the “loaded” filters in a freezer (−18 °C or less) if not processed immediately.

#### Particulate fraction

##### Collection of particulate matter from filters

(1) Thaw frozen filters prior to scraping if these were not processed immediately.
(2) Place the filter on a smooth surface (e.g., Plexiglas) and scrape using a Teflon scraper.
(3) Use P-free growth medium (sparingly) to facilitate particulate matter scraping, suspension and removal, rinsing the collection (slurry) into a glass bottle like a Boston round bottle.
(4) Store the bottle with the slurry (max 1/3 full) frozen at a 45° angle (to increase surface area of water/air interface to prevent breakage) until required for analysis.

##### Measurement of sample P richness

The PP concentration (µgP/L) of the sample was measured in *Pre-Sampling* as guidance for determining the volume to be filtered to yield a slurry for the particulate phase bioassay. Here, sample P richness (µgP/mgDM) is the mass of P per total sample mass and is measured to determine the volume of slurry that, when added to the bioassay chamber, will achieve a P content of 500–1000 µgP. As with the *Pre-Sampling* PP analysis, the slurry PP analysis only provides guidance for slurry addition to the assay; the PP content of the dark chamber will be determined at the start of the bioassay.

(1) Thaw frozen slurry slowly to prevent bottle breakage if slurry was not processed immediately
(2) Combine slurry from all glass bottles from *Collection* in an Erlenmeyer flask and mix thoroughly (e.g., using a magnetic stir bar and a stir plate).
(3) Pipette 5 mL of slurry onto a pre-weighed aluminum weighing tin and perform a dry mass analysis, yielding the dry mass (DM, mg) of the 5 mL of slurry.
(4) Perform a TP analysis on another 5 mL subsample of slurry, yielding the mass of P (µgP) contained in the 5 mL subsample. TP analysis is used here to capture any P that has been desorbed from particulate to the liquid phase.
(5) Divide the mass of P (µgP) in the slurry (Step 3) by the mass of DW solids in the slurry (Step 2) to calculate P richness (µgP/mgDM).

##### Particulate assay apparatus setup

**Fig. 1:**
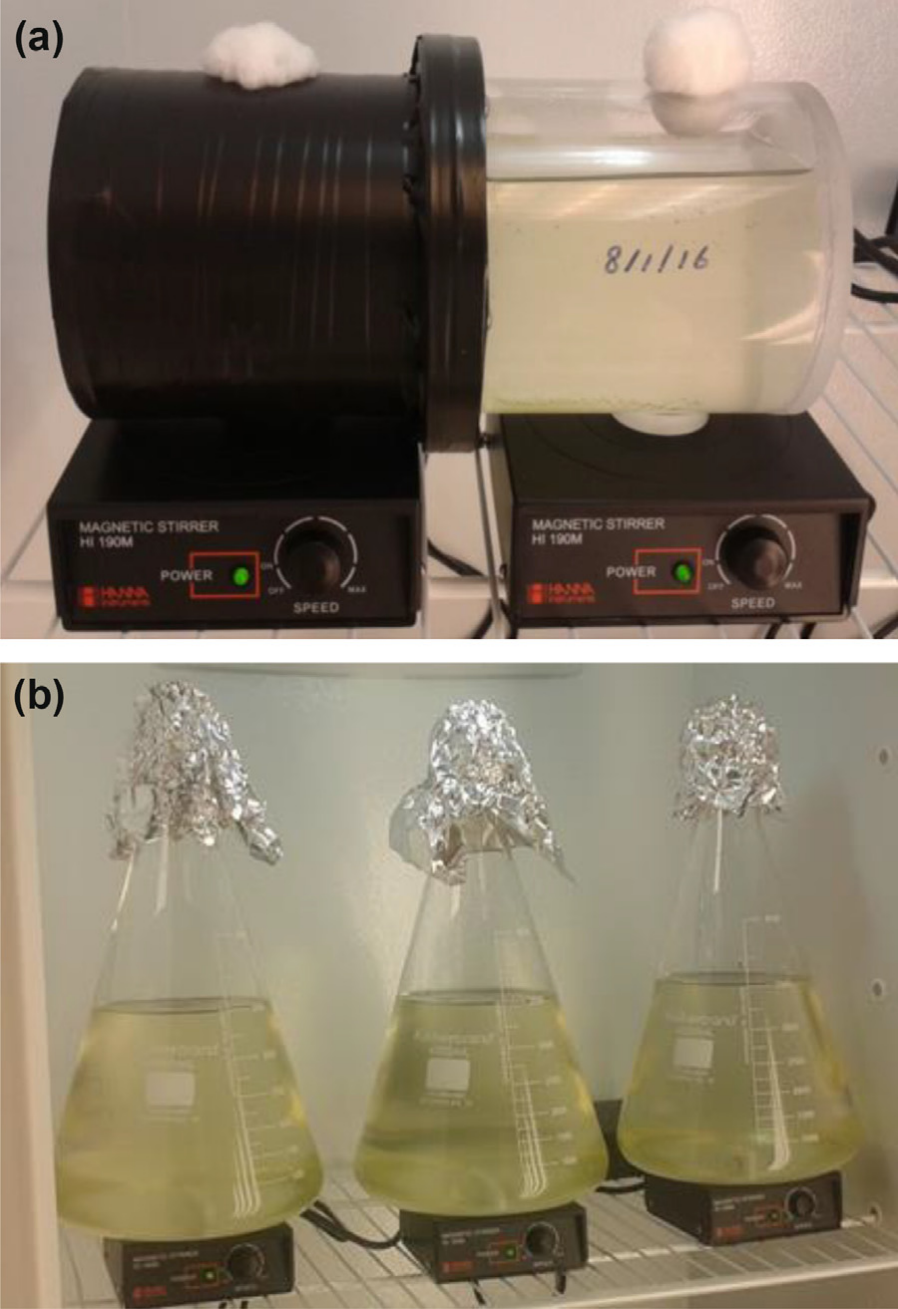
(a) DCDA apparatus setup for particulate assay. (b) Soluble fractionation apparatus setup.

The particulate phase bioassay is performed in a dual culture diffusion apparatus (DCDA, Fig. 1a; [7]). A DCDA is a small cylindrical vessel divided into two chambers and can be built from Plexiglas or custom blown from glass by Bellco Glass. One chamber is darkened to exclude light and receives the sample slurry; the other is open to light and receives the assay algae. The apparatus is placed in an incubator (20 °C and 600 µmol m^−2^ s^−1^ of light). The chambers are separated by a black, 0.45 µm membrane filter (e.g., Pall S80677) and a magnetic stir bar is placed in each chamber. Each DCDA is placed on two stir plates for continuous mixing in each chamber. Each chamber has a port, lightly fitted with glass wool, to provide for particulate-free gas exchange, slurry and algae addition and algae sample withdrawal. It is imperative that the apparatus does not leak and filters do not tear. As sample PP in the dark side is released by desorption and mineralization, the resultant soluble P diffuses to the light side and the bioavailable fraction is taken up by the assay algae. The light chamber is harvested and re-stocked with P-starved algae after each incubation interval.

##### Startup

Initial filling of the dark chamber

(1) Based on the P richness of the sample (method above), determine the volume of slurry needed to yield 500–1000 µgP in the dark side chamber at startup.
(2) Add the desired volume of slurry to a 1 L volumetric flask.
(3) Dilute the slurry to 1 L with P-free growth medium (macro nutrient stock solutions A and C and trace metal stock solution) and mix well.
(4) Pour 800 mL of the diluted slurry into the DCDA dark chamber, retaining the balance of diluted slurry for determination of the initial P content of the dark chamber.
(5) Measure PP of three 50 mL subsamples of the retained diluted slurry to determine the mass of P initially added to the dark chamber (*P_dark,initial_*).

Initial filling of the light chamber

(1) Fill 6 centrifuge tubes with 50 mL of P-starved algal culture.
(2) Centrifuge the culture at 3500 rpm for 5 min, clearing the supernatant of algae and forming an algal pellet at the bottom of the tube.
(3) Carefully pour off the supernatant (leaving ~15 mL in tube) and resuspend the algal pellet in P-free growth medium to a total volume of 50 mL.
(4) Centrifuge again (to remove any carryover of dissolved phosphorus), pour off the supernatant (leaving ~ 15 ml in tube) and resuspend to a total volume of 50 mL in P-free growth medium.
(5) Add the contents of 3 tubes to P-free growth medium, achieving a final volume of 800 mL and add this to the DCDA light chamber, taking care to avoid spillage.
(6) Filter the contents of the remaining 3 tubes (0.45 µm membrane filter) and measure PP on the filtride to determine the mass of P initially added to the light chamber (*P_light,initial,t__=__0_*).
(7) Prior to startup, check the DCDA for leaks, condition of ports and operation of stir bars.
(8) Periodically check light, temperature, operation of stir bars and condition of ports (glass wool).

##### Harvest, restocking and initial condition of the light chamber

At the conclusion of each nominal assay interval (ideally every 2–3 days, i.e., *t* = 2, 4, 7, 11, 16, 21 and 27; or until uptake ceases), the algae are harvested from the light chamber, the exact time (*t*) is noted and PP is measured to determine the mass of bioavailable P generated in the dark chamber and subsequently taken up by the algae in the light chamber. The algae harvested on that day are replaced (restocked) with fresh P-starved algae.

Harvest

(1) Carefully remove the entire 800 mL volume from the light chamber (a large syringe with a piece of tubing or a peristaltic pump works well) and transfer to a 1 L graduated cylinder, ensuring that the algae are completely removed by swirling the liquid. Take care so as not to puncture the membrane filter separating the chambers.
(2) Distribute the 800 mL volume harvested from the light chamber to 16 centrifuge tubes (50 mL each) and centrifuge at 3500 rpm for 5 min.
(3) Pour the supernatant from all 16 centrifuge tubes back into the graduated cylinder, retaining the algae pellets in the centrifuge tube with ~15 ml supernatant.
(4) Resuspend the algae pellets in the remaining supernatant and filter through a 0.45 µm membrane filter.
(5) Add the filtrate to the graduated cylinder, retaining the filtride. At the final harvest, save the filtrate for soluble phase P analysis (for mass balance determination).
(6) Measure PP on the filtride to determine the mass of particulate P present in the light chamber at harvest (*P_light,final,t_*).

Restocking

(1) Add the algae from 150 mL (3 centrifuge tubes) of P-starved culture, prepared according to Steps 2–4 of the *Initial filling of the light chamber* procedure, to the graduated cylinder.
(2) Gently add P-free growth medium to the graduated cylinder to bring the volume to 800 mL, compensating for loss due to evaporation. Return the contents of the graduated cylinder to the light chamber and place the DCDA back in the incubator.
(3) Check light, temperature, operation of stir bars and condition of ports (glass wool).

Initial Condition

(1) Filter 150 mL (3 centrifuge tubes) of the P-starved algal culture used to restock the light chamber as described in *Initial filling of the light chamber*, Steps 2–4.
(2) Measure PP on the filtride to determine the mass of particulate P initially present in the light chamber for this incubation interval (*P_light,initial,t_*).

##### Calculation of particulate phase bioavailability

The assay is concluded once increases in bioavailable P have ceased and the total amount of P taken up (an asymptote) has been reached. The mass of bioavailable P generated by the sample particulate phase is determined for each discrete assay interval as

(2) $$P_{bio,PP,t}=\;P_{\;light,PP,final,t}-\;P_{\;light,PP,initial,t}$$

where

*P*_*bio,*__*PP,*__*t*_ = mass of bioavailable P generated in the dark chamber and taken up in the light chamber over assay interval *i*, µgP
*P*_*light,*__*PP,*__*initial,*__*t*_ = mass of P initially present in the light chamber, µgP
*P*_*light,*__*PP,*__*final,*__*t*_ = mass of P finally present in the light chamber, µgP

After the final harvest, obtaining *P_bio,PP,final,t_*, the total (cumulative) bioavailable PP of the sample is calculated by summing all *P_bio,PP,t_* obtained for each sampling interval:

(3) $$P_{bio,PP}=\;\sum_{t=0}^{t=t\;final}P_{bio,PP,t}$$

where

*P*_*bio,*__*PP*_ = cumulative mass of P generated in the dark chamber and taken up by the assay organism over the entire incubation, µgP

**Fig. 2:**
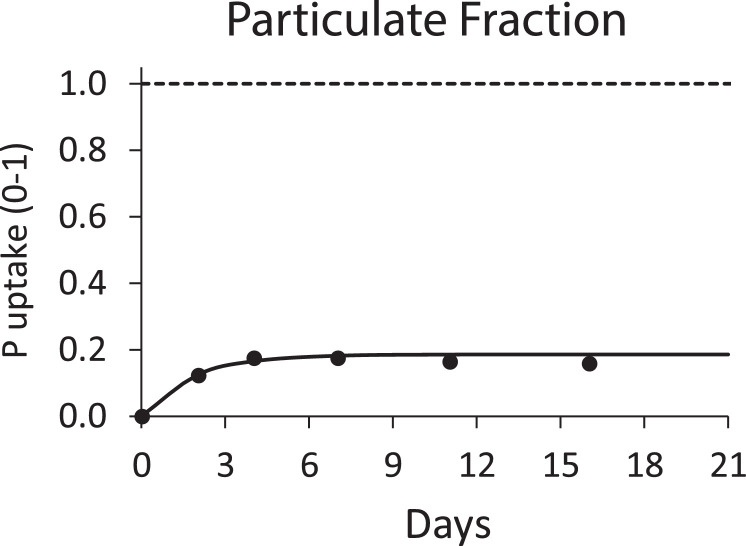
Normalized uptake of phosphorus in a particulate fractionation experiment. The dashed horizontal line represents total phosphorus initially present in the system, the solid line represents modeled uptake, and the solid dots indicate measured uptake (bioavailable fraction is 19%).

The results may be presented as a time series of *P_bio,PP,t_* (Fig. 2).

The bioavailable fraction of the particulate phase sample is equal to the cumulative mass of bioavailable P harvested from the light chamber at the end of the assay divided by the mass of P added to the dark chamber at the start of the assay (see *Initial Filling – Dark Chamber*),

(4) $$f_{bio,PP}=\frac{P_{bio,PP}}{P_{dark,PP,initial}}$$

where

*f*_*bio,PP*_ = particulate P fraction bioavailable, dimensionless
*P*_*dark,PP,initial*_ = mass of P added to the dark chamber at the start of the assay, µgP

##### Closure

Closure on the bioassay is determined by performing a phosphorus mass balance at the time of the final harvest.

(1) Measure the volume contained in the light chamber and perform triplicate SRP and TDP analyses to determine the final mass of SRP (*P_light,SRP,final_*) and DOP (*P_light,DOP,final_*).
(2) Measure the volume contained in the dark chamber and perform triplicate TP analyses to determine the final mass of P in the dark chamber (*P_dark,TP,final_*).

The initial mass of P in the dark chamber is compared to the sum of the final mass of P in the dark chamber plus the final masses of SRP and DOP in the light chamber plus the cumulative mass of P harvested from the light chamber over the course of the assay:

(5) $$P_{dark,intial}=P_{dark,final}+P_{light,SRP,final\;}+P_{light,DOP,final\;}+P_{bio,PP}$$

Closure of the mass balance (total P at the beginning of the assay = total P at the end of the assay) is considered achieved when the sum of P fractions (right side of Eq. (5)) is >90% of *P*_*dark, intial*_.

##### Kinetics determination

The rate constant (*k_bio,PP_*) for transformation of sample particulate P to bioavailable P (taken up in the light side) is determined by plotting a time series of *P_bio,PP,t_* (using actual harvest times in place of the ‘*t*’ identifier) and fitting the results to a first order accumulation function (Fig. 2),

(6) $$P_{bio,PP,t}=P_{bio,PP}\cdot \left(1-e^{-k_{bio,PP}\cdot t}\right)$$

where

*P*_*bio, PP, t*_ = mass of bioavailable P taken up at time t, µgP
*k*_bio,PP_ = rate constant for generation of bioavailable P from particulate P, (d^−1^)
*t* = time (d)

Values of *k_bio,PP_* are presented for multiple systems [see: 1,2,8,10,12].

#### Soluble fraction

The soluble fraction assay is an adaptation of the Algal Assay Bottle Test [13], which uses *Selenastrum capricornutum* as the assay organism (see *Algal Culture* above). The assay is performed on the water sample filtrate (see *Sample filtration* above) incubated in a glass vessel (Fig. 1b). The bioavailable portion of the soluble phase is determined by measuring the drawdown of SRP and DOP (calculated from paired measurements of SRP and TDP). The observed drawdown may be transformed to express results as an accumulation as for the particulate phase assay; see also [8]).

As the soluble phase sample cannot be concentrated as in the particulate assay, measured P concentrations are lower in the soluble assay and constrained to the detection limits of the SRP/TDP procedures (in our laboratory, 0.2 µgP/L). Harvesting scheduled for Days 1, 2, 4, 7, 10 and 14 captures drawdown well, with SRP depleted in 1*–*2 days and bioavailable DOP exhausted in 6*–*10 days. The experiment may be terminated when the drawdown curve reaches an asymptote.

##### Startup

(1) Measure SRP and TDP in triplicate aliquots of the sample filtrate (see *Sample filtration*) to establish the initial soluble P concentration (*P_SRP,initial_* and *P_DOP,initial_*).
(2) Prepare 150 mL of P-starved algae culture as described in Steps 1*–*4 of the *Initial Filling – Light Chamber* section of the particulate phase assay.
(3) Add 1 L of filtrate and the assay algae (Step 2) to a glass vessel (a 4 L Erlenmeyer flask works well) and dilute to 2 L (a volume that accommodates 7–10 sampling events) leaving at least 50% of head space.
(4) Collect samples from the vessel in triplicate and measure TP to represent the initial condition in the mass balance closure analysis.
(5) Incubate the container under light-saturating conditions (600 µE/m^2^/s) at 20 °C, with the solution well-mixed (using a stir bar and stir plate), aerated (using an aquarium bubbler) and with an in-line glass wool filter to plug the flask.

##### Harvest

(1) Collect triplicate samples for SRP and TDP determination on nominal Days 1, 2, 4, 7, 10 and 14 and record exact time (*t*).
(2) Repeat harvest until the concentration versus time curve reaches an asymptotic (~2 days for SRP and ~10 days for DOP).

##### Calculation of soluble phase bioavailability

**Fig. 3:**
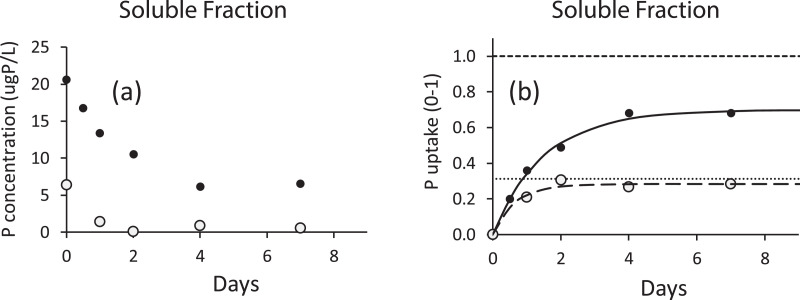
(a) Depletion curve of SRP and TDP over time. (b) Normalized uptake of phosphorus in a soluble fractionation experiment. Solid points represent measured TDP uptake, the solid line represents modeled TDP uptake, and the horizontal dashed line above it represents the amount of TDP initially in the system. Open circles represent measured SRP uptake, the large dashed line represents the model fit, and the horizontal dotted line represents the total amount of SRP initially in the system (bioavailable SRP fraction is 98%, TDP is 70% and DOP is 58%).

The concentrations of SRP (*P_SRP,t_*) and DOP (*P_DOP,t_*) in the vessel are measured at each discrete assay interval. Because the soluble phase assay is performed with no restocking of starved algae, here, concentration is used in place of mass as in the particulate phase assay. Concentrations may be plotted as a function of time to illustrate SRP and DOP depletion (Fig. 3a). A cumulative time series (Fig. 3b) may also be developed to illustrate the accumulation of bioavailable P in the same manner as for bioavailable particulate P:

(7) $$P_{bio,SRP,t}=\;P_{SRP,initial}-P_{\;SRP,t}$$

where

*P*_*bio,*__*SRP,*__*t*_ = bioavailable SRP at time *t*, µgP/L
*P*_*SRP,*__*initial*_ = initial SRP, µgP/L
*P*_*SRP,*__*t*_ = SRP at time t, µgP/L

with a special case of *P_bio,SRP,t_* being that for the final harvest, *P_bio,SRP,t,final_*, representing the total bioavailable SRP of the sample.

The bioavailable SRP fraction of the soluble phase sample is equal to the total bioavailable SRP concentration divided by the initial SRP concentration.

(8) $$f_{bio,SRP}=\frac{P_{bio,SRP,final}}{P_{SRP,initial}}$$

where

*f*_*bio, SRP*_ = SRP fraction bioavailable, dimensionless

The calculation is performed in an identical fashion for the DOP analyte. Results for SRP and DOP may be added together to yield *P_bio,soluble_* and to calculate *f_bio,soluble_* using Eq. (8) and substituting the sums of SRP and DOP as *P_bio,final_* and *P_initial_*.

*Closure*

Closure for the soluble phase assay is achieved by taking triplicate samples from the vessel and determining the final TP concentration. Comparison to the initial TP in the vessel provides an estimate of closure, for which >95% can be obtained (i.e., when TP final is within 5% of initial TP concentration).

##### Kinetics determination

The SRP component of dissolved P is known to be fully and freely available. This means that *f_bio,SRP_* → 1 and that uptake occurs at a time scale of minutes with any manifestation of a (time-variant) kinetic behavior likely an artifact of the balance between the sample SRP concentration and the uptake capacity of the assay organism standing crop. The DOP fraction is typically not fully bioavailable and exhibits a (time-variant) kinetic behavior as it is converted enzymatically to SRP and taken up by the assay organism. The kinetics of DOP bioavailability may be characterized as a depletion curve fit to first order function that includes a refractory component (the asymptote of the curve; Fig. 3a),

(9) $$P_{\mathrm{DOP},t}=P_{\mathrm{bio},\mathrm{DOP},t,\mathrm{final}}\cdot \left(e^{-k_{\mathrm{bio},\mathrm{DOP}}\cdot t}\right)+P_{\mathrm{refr}\mathrm{acto}\mathrm{ry},\mathrm{DOP}}$$

where

*k*_bio,DOP_ = rate constant for generation of bioavailable P from DOP, (d^−1^)
*P*_*refractory, DOP*_ = refractory P concentration, µgP/L

Although the assay is performed by tracking depletion, results may be represented as a bioavailable DOP accumulation, mirroring the form presented for the particulate phase assay (Eq. (4)),

(10) $$P_{\mathrm{bio},\mathrm{DOP},t}=P_{\mathrm{bio},\mathrm{DOP},t,\mathrm{final}}\cdot \left(1-e^{-k_{\mathrm{bio}}\cdot t}\right)$$

### Bioavailable fraction of total phosphorus

The TP fraction bioavailable (*f_bioTP_*) may be determined based on the measured concentrations of PP, SRP and DOP in the sample and their respective *f_bio_* values:

(11) $$f_{bio,TP}=\;\frac{f_{bio,SRP}\cdot SRP+f_{bio,DOP}\cdot DOP+f_{bio,PP}\cdot PP}{TP}$$

### Method validation

The use of a high-pressure filtration apparatus and filter scraping are likely to increase the fraction of bio P as algal cells in the sample may become damaged (aiding P-release). Measurements of the fraction of bio P may thus be seen as a conservative estimate. Bias in the method will not impact results when comparing bio P between systems or over time as they describe the relative difference.

Replication of results for the soluble and particulate phase assays is good. Assays performed on triplicate samples collected from the Fox River, WI and Lake Winnebago, WI yielded a coefficient of variation for TDP and SRP of 7.7 and 6.3%, respectively. Comparable results were obtained [11] in assays performed on samples collected from the Maumee River, OH with coefficients of variation of 6 and 5%, respectively.

### Typical results

The figures shown below show the typical results for the particulate and soluble fractionation experiments where uptake is highest in the initial days of the experiments and slows down until uptake ceases.

(1) Bioassay vessels: (a) particulate phase and (b) soluble phase.
(2) Particulate phase accumulation.
(3) Soluble phase (a) depletion and (b) accumulation.
